# Application of bedside ultrasound in predicting the outcome of weaning from mechanical ventilation in elderly patients

**DOI:** 10.1186/s12890-021-01605-4

**Published:** 2021-07-09

**Authors:** Shigang Li, Zhe Chen, Weifeng Yan

**Affiliations:** Department of Respiratory and Critical Care Medicine, Beijing Haidian Hospital, No. 29, Zhongguancun St, Haidian District, Beijing, 100080 China

**Keywords:** Ultrasonography, Mechanical ventilation, Weaning, Diaphragm excursion, Diaphragm thickening fraction

## Abstract

**Background:**

With the increased ageing of society, more and more elderly people are admitted to the intensive care unit, How to accurately predict whether elderly patients can successfully wean from the ventilator is more complicated. Diaphragmatic excursion (DE) and diaphragm thickening fraction (DTF) were measured by bedside ultrasound to assess diaphragm function. The lung ultrasound score (LUS) and the rapid shallow breathing index (RBSI) were used as indices of diaphragm function to predict the outcome of weaning from mechanical ventilation. The aim of this study was to examine the clinical utility of these parameters in predicting extubation success.

**Methods:**

This prospective study included 101 consecutive elderly patients undergoing a trial of extubation in the ICU of Haidian Hospital between June 2017 and July 2020. Patients were divided into the successful weaning group (n = 69) and the failed weaning group (n = 32). Baseline characteristics, including RSBI, were recorded. Measurements of DE, DTF and LUS were made using ultrasound within 24 h before extubation.

**Results:**

Median DE was greater in patients with extubation success than in those with extubation failure (1.64 cm vs. 0.78 cm, *p* = 0.001). Patients with extubation success had a greater DTF than those with extubation failure (49.48% vs. 27.85%, *p* = 0.001). The areas under the receiver operating curves for the RSBI, LUS, DE and DFT were 0.680, 0.764, 0.831 and 0.881, respectively. The best cut-off values for predicting successful weaning were DTF ≥ 30%, DE ≥ 1.3 cm, LUS ≤ 11, and RSBI ≤ 102. The specificity of DTF (84%) in predicting weaning outcome was higher than that of RBSI (53%), that of LUS (55%), and that of DE (62%). The sensitivity of DTF (94%) was greater than that of RBSI (85%), that of LUS (71%), and that of DE (65%). The combination of RSBI, LUS, DE, and DTF showed the highest AUC (AUC = 0.919), with a sensitivity of 96% and a specificity of 89%.

**Conclusions:**

DTF has higher sensitivity and specificity for the prediction of successful weaning in elderly patients than the other parameters examined. The combination of RSBI, LUS, DE and DFT performed well in predicting weaning outcome. This has potentially important clinical application and merits further evaluation.

## Introduction

With the increased ageing of society, more and more elderly people are admitted to the intensive care unit, which will become the focus and a challenge of the medical system. Therefore, the problem of elderly patients is worthy of attention.

Mechanical ventilation is an essential life support technology for many critically ill patients [1, 2]. Appropriate timing of extubation is crucial for weaning critically ill patients from invasive mechanical ventilation. Extubation failure may lead to respiratory failure or reintubation, which may worsen the prognosis [3]. Delayed extubation may lead to ventilator-associated pneumonia, contributing to a poor prognosis. Successful weaning reduces the morbidity and mortality of patients when their primary disease improves [4]. Therefore, accurate prediction of weaning outcome is very important. However, choosing the appropriate time for extubation remains a challenge to intensivists caring for critically ill patients. Furthermore, while several parameters have been employed in deciding when to extubate, all have limitations [5].

The diaphragm is the primary muscle of inspiration used in spontaneous breathing, so assessment of diaphragm dysfunction is pivotal in patients undergoing weaning or a trial of extubation [6]. Ultrasonography (US) allows the qualitative and quantitative assessment of diaphragmatic function in terms of diaphragmatic thickness (DT) and diaphragmatic amplitude during contraction, which are helpful clinically in diagnosing diaphragmatic weakness and in examining respiratory workload in patients in the ICU. Diaphragmatic US and other measurements, such as the rapid shallow breathing index (RSBI) and the lung ultrasound score (LUS), have been used to predict successful weaning from mechanical ventilation or extubation in the intensive care unit (ICU) [7].

Muscle fatigue or weakness is one of the main factors in this success. As the main muscle of human respiration, the diaphragm plays an important role in the pathophysiological process of respiratory failure. In the past, the RSBI and other indicators were used for evaluation, but data on muscle motor function were lacking. In recent years, with the application of advanced ultrasound technology, increasing attention has been given to ultrasound evaluation of diaphragm function, including diaphragm thickening fraction (DTF), diaphragm displacement (DE) and lung ultrasound score (LUS). These indicators can be used to evaluate the alveolar ventilation insufficiency caused by alveolar collapse in critically ill patients during weaning, and they have certain predictive value for the weaning results of patients on mechanical ventilation.

Due to the pathophysiological changes that come along with old age, the function of the diaphragm and corresponding respiratory muscle changes to a certain extent, making them weaker than those of young people. Pulmonary function begins to decline at approximately age 25 years. In healthy non-smoking individuals, spirometric measures forced expiratory volume in 1 s (FEV1) and forced vital capacity (FVC) decrease by 30 mL per year in men and 23 mL per year in women, with accelerated loss after age 65 year [8, 9], Respiratory muscle strength decreases with age. Maximal effort trans diaphragmatic pressure gradients in older individuals are lower than in younger subjects, reflecting decreased diaphragmatic strength [10]. In one study, diaphragm strength in the elderly was 13% less than in a younger group by maximal sniff. Other measurements have shown 25% lower diaphragmatic strength in the elderly [11]. The weaning outcome of the mechanical ventilation process for elderly patients is more difficult and complicated. How to more accurately predict whether elderly patients can successfully wean from the ventilator is very important to clinical work. In elderly patients weaning off of mechanical ventilation, there is a lack of relevant research on the predictive value of ultrasound indicators of respiratory muscle function and their role, if any, in the evaluation. In the past, ultrasound was used to evaluate diaphragm function and predict the success or failure of extubation. However, the pathophysiology of elderly patients over 65 years old has changed. It is worth studying which indicators are more suitable for them and what their effects are. Mechanical ventilation is a common means of life support for critically ill patients, and successful weaning is an important part. At present, the greatest difficulty in the weaning process is the lack of one or more gold-standard indicators to accurately predict the success of weaning.

The aim of this study was to examine the clinical utility of these diaphragmatic and other parameters in predicting extubation success. Through this research, we hoped to make it clear which indicators are more suitable for elderly patients and how strong their predictive value is. Identifying such indicators will better guide clinical work, reduce mortality and improve the prognosis of patients, making them worthy of clinical promotion.

## Material and methods

This study was conducted at the ICU of Haidian Hospital, Beijing. The study was approved by the Ethical Clearance Committee of Haidian Hospital. We enrolled consecutive adult patients aged > 65 years who were admitted to the ICU of Haidian Hospital between June 2017 and July 2020. All patients and their families signed informed consent forms.

The inclusion criteria were as follows: (1) age ≥ 65 years, (2) respiratory failure requiring mechanical ventilation for more than 24 h, and (3) meeting all of the following criteria for the SBT: FiO_2_ < 50%, positive end-expiratory pressure ≤ 5 cmH_2_O, respiratory rate ≤ 30 breaths/min, PaO_2_/FiO_2_ > 200 mmHg, and haemodynamic stability in the absence of vasopressors [8]. The exclusion criteria were neuromuscular disease, diaphragmatic paralysis and tracheostomy. Patients with diaphragm paralysis on either side were excluded. Each patient’s condition was evaluated, and they entered the study only when their underlying disease was under control and they met the above criteria. All patients’ cough reflex had recovered, and they had a powerful cough. They had a certain ability to clear their airway secretions.

Patients were divided into the successful weaning group and the failed weaning group. The clinical team administered the spontaneous breathing test (SBT) according to the patient's condition, and the research team collected the data. Before extubation, ultrasound examination was performed to evaluate diaphragm function and record the data. The SBT was performed when the patient met the standard above. In the spontaneous breathing test, patients were given T-tube oxygen inhalation at 5 L/min and were observed for half an hour. We took one measurement for each patient in the SBT. During SBT, ultrasound examination was performed to evaluate diaphragm function and record the data. The ultrasound examination protocol was standardized. Ultrasound examination was performed immediately at the beginning of SBT.

Extubation was performed if all of the following criteria were satisfied over a period of 30 min: good tolerance of the SBT, respiratory rate < 35 breaths/min, heart rate < 120 beats/min or heart rate variability ≤ 20%, oxygen saturation ≥ 90%, 80 mmHg < systolic blood pressure < 180 mmHg or < 20% change from baseline and absence of increased breathing work or distress signs [12]. After the patient passed the SBT, extubation was performed offline. Patients with successful extubation were included in the success group, and the others were included in the failure group.

### Definition of extubation success or failure

Extubation success was defined as sustained spontaneous breathing for more than 48 h following extubation without noninvasive positive-pressure ventilation (NPPV) or invasive ventilation. Extubation failure was defined the need for reintubation, the application of NIV within 48 h or the need for tracheostomy [13]. Because the application of noninvasive ventilation indicates that patients still need mechanical ventilation support, it is always attempted first in weaning. If extubation failed, after the patient's condition improved, the patient was evaluated again, and SBT and extubation were attempted again when the standard was met.

### Measurements

#### Clinical data collection

The following baseline characteristics were recorded: age, sex, body mass index, primary disease, length of stay in the ICU, duration of mechanical ventilation, acute physiology and chronic health evaluation II (APACHE II), sequential organ failure assessment (SOFA) and the rapid shallow breathing index (RSBI). Ultrasound was performed by two different experienced doctors.

#### Lung ultrasound

The lung ultrasound score (LUS) was calculated according to the observed worst ultrasound pattern: normal aeration, LUS = 0; moderate loss of lung aeration (multiple, well-defined B lines), LUS = 1; severe loss of lung aeration (multiple coalescent B lines), LUS = 2; lung consolidation, LUS = 3. Finally, the LUSs of all the parts were added to obtain the total LUS for each patient (maximum 36 points) [14].

#### Evaluation of diaphragm function

Diaphragm movement was measured with a 2–5 MHz probe (Mindray) placed over one of the lower intercostal spaces in the right anterior axillary line for the right diaphragm and the liver, serving as an acoustic window. Two-dimensional (2D) mode was used to search for the line of the right hemidiaphragm. The right hemidiaphragm was imaged at the zone of apposition of the diaphragm and rib cage on the mid-axillary line between the 8th and 10th intercostal spaces [15]. Diaphragm thickness (DT) was measured at both end-inspiration and end-expiration using a 6–13 MHZ linear US probe set to B mode (M-Turbo) [18, 19].

DT difference (DTD) was calculated by subtracting DT at end-expiration from DT at end-inspiration. Diaphragm thickening fraction (DTF) was calculated as: DTD/(DT at end-expiration) × 100% [16].

Diaphragmatic excursion (DE) was identified and measured. DE amplitude was measured on the vertical axis by tracing from the baseline to the point of maximum height of inspiration on the graph [17].

### Statistical analysis

The sample size needed was calculated using the Epi-Info software statistical package created by the World Health Organization and Centers for Disease Control and Prevention, Atlanta, Georgia, USA, version 2002. The sample size was calculated at N = 64 based on the 95% confidence interval.

Data are presented as median and interquartile range (IQR) or mean ± standard deviation for continuous variables and number (%) for noncontinuous variables. The chi-square test or Fisher’s exact test was used to compare categorical variables, and the independent sample t test or Mann–Whitney U test was used to compare continuous variables between the two groups. Logistic regression analysis was used to evaluate the correlation between the ultrasound diaphragm function evaluation index and SBT success or failure as well as weaning success or failure. The sensitivity and specificity were calculated for the parameter index to predict extubation success. Receiver operating characteristic (ROC) curves were used to evaluate and compare the RSBI, LUS, DE and DTF for extubation success. A logistic regression model was used to fit multiple joint diagnostic or predictive indicators to form new joint predictors. Construction of the joint prediction model with predictive indicators as classified variables: Taking whether the patient weaned successfully from the ventilator as a binary outcome variable and taking DTF, DE, LUS, and RSBI as covariates, a logistic regression equation was established to generate a joint predictor for judgement. Generation of joint predictor and determination of the optimal critical value: The original covariates were fitted to the new joint predictor by using the predictor command. The sensitivity, specificity and prediction accuracy of the combined predictor were determined. When the Youden index was at its maximum, the value corresponding to the new joint predictor was the best critical value. A receiver operating characteristic curve (ROC) was constructed. The area under the ROC curve (AUC) of the joint predictors was compared with that of the original indicators to determine the best critical value. Sensitivity, specificity and predictive accuracy were calculated. A *p* value < 0.05 was considered statistically significant. The data were analysed using SPSS version 20.0.

## Result

This prospective study included 101 consecutive elderly patients undergoing a trial of extubation in the ICU of Haidian Hospital between June 2017 and July 2020. Patients were divided into the successful weaning group (n = 69) and the failed weaning group (n = 32). A wide variety of diagnoses, comorbidities, and population characteristics were represented in the patients studied (Table 1).

**Table 1:** Comparison of baseline characteristics in patients with extubation success and failure

Variable	Extubation success (n = 69)	Extubation failure (n = 32)	*p* value
Age, years (IQR)	70 (65–87)	68 (66–89)	0.760
Male sex (%)	35 (74.5)	7 (53.8)	0.181
Body mass index, kg/m^2^ (IQR)	21.4 (19.0–24.9)	22.3 (20.2–23.5)	0.859
Simplified acute physiology score III (IQR)	71 (63–77)	59 (51–81)	0.190
APACHE II	14.5 (10–22)	15.2 (11–24)	0.18
SOFA score (x ± s) on admission	4.75 ± 2.14	5.13 ± 2.25	0.188
SOFA score (x ± s) on SBT	3.84 ± 1.24	4.25 ± 1.65	0.158
Clinical frailty score	5.11 ± 1.8	5.23 ± 1.9	0.512
Primary disease			
Pulmonary infection (N, %)	28 (40.58)	10 (31.25)	0.625
AECOPD (N, %)	7 (10.1)	4 (12.5)	0.246
Acute left heart failure (N, %)	3 (4.3)	1 (3.1)	0.346
Intracranial lesions (N, %)	10 (14.5)	6 (18.75)	0.324
Complication			
Respiratory failure (N, %)	45 (65.2)	20 (62.5)	0.821
ARDS (N, %)	6 (8.7)	2 (6.25)	0.111
PaO_2_/FiO_2_ = 201–300 mmHg (mild ARDS)	2 (2.9)	0 (0)	0.50
PaO_2_/FiO_2_ = 101–200 mmHg (moderate ARDS)	2 (2.9)	1 (3.1)	0.63
PaO_2_/FiO_2_ ≤ 100 mmHg (severe ARDS)	2 (2.9)	1 (3.1)	0.63
Shock (N %)	32 (46.38)	18 (56.25)	0.725
Obstruction of sputum drainage (N, %)	24 (34.8)	12 (37.5)	0.526
Total duration of mechanical ventilation in hospital (IQR)	6 (3–10)	10 (6–14)	0.001
ICU stay time (IQR)	8 (5–16)	12 (6–21)	0.0001
Total length of stay (d, x ± s)	19.73 ± 3.69	24.67 ± 4.12	0.068

### Comparison of baseline characteristics, LUS, RSBI, DE, and DTF in patients with extubation success versus failure

The causes of mechanical ventilation in the ICU included pneumonia, COPD, ARDS, shock, heart failure, etc. The specific proportions are shown in Table 1. There was no significant difference in age, sex, SOFA score, basic diseases, and complications between the two groups. In the failed weaning group, 9 patients were reintubated, and the remaining 23 received NPPV. The length of ICU stay and duration of mechanical ventilation during ICU stay in patients who failed to wean were both significantly greater than those in successfully weaned patients (*p* < 0.05, Table 1). The LUS and RSBI in the failed weaning group were significantly greater than those in the successful weaning group. Furthermore, the diaphragm function indices (DE, DTF) were significantly lower in the failed weaning group than in the successful weaning group (*p* < 0.05, Table 2).

**Table 2:** Comparison of LUS, RSBI and diaphragm parameters (DE, DTF) in patients with extubation success and failure

Variables	Successful weaning group (n = 69)	Failed weaning group (n = 32)	*p* value
RSBI (breaths/min/L)	75.68 ± 18.36	105.29 ± 16.07	0.003
LUS	11 (7–14)	15(11–20)	0.025
DE (cm)	1.65 (1.23–1.91)	0.80 (0.51–1.1)	0.001
DTF (%)	49.48 ± 12.50	27.83 ± 9.95	0.001

*DE* diaphragmatic excursion, *DTF* diaphragm thickening fraction, *RSBI* rapid shallow breathing index, *LUS* lung ultrasound score

### Multivariate logistic regression for risk of weaning failure

Multivariate logistic regression revealed that RSBI (OR = 1.673, *p* = 0.03), LUS (OR = 1.736, *p* = 0.001), DE (OR = 3.942,* p* = 0.014), and DTF (OR = 1.203, *p* = 0.001) were significantly associated with an increased risk of weaning failure (Table 3).

**Table 3:** Multivariate logistic regression for risk of weaning failure

Variables	Odds ratio	CI = 95%	*p*
RSBI	1.673	0.821–3.403	0.03
LUS	1.736	1.335–2.372	0.001
DE	3.942	1.423–19.943	0.014
DTF	1.203	1.223–14.851	0.001

*CI* confidence interval, *DE* diaphragmatic excursion, *DTF* diaphragm thickening fraction, *RSBI* rapid shallow breathing index, *LUS* lung ultrasound score

### Predictive accuracy of diaphragm indices for weaning outcome

**Fig. 1:**
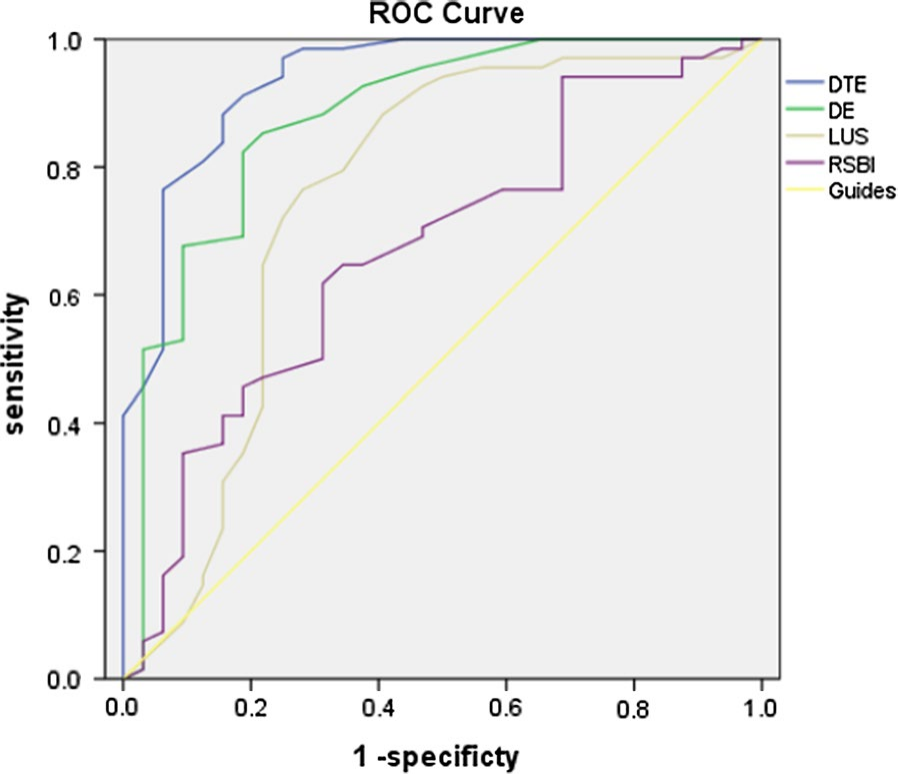
Receiver operating characteristic curves for DTF, DE, LUS, and RSBI. *DE* diaphragmatic excursion, *DTF* diaphragm thickening fraction, *RSBI* rapid shallow breathing index, *LUS* Lung ultrasound score

**Fig. 2:**
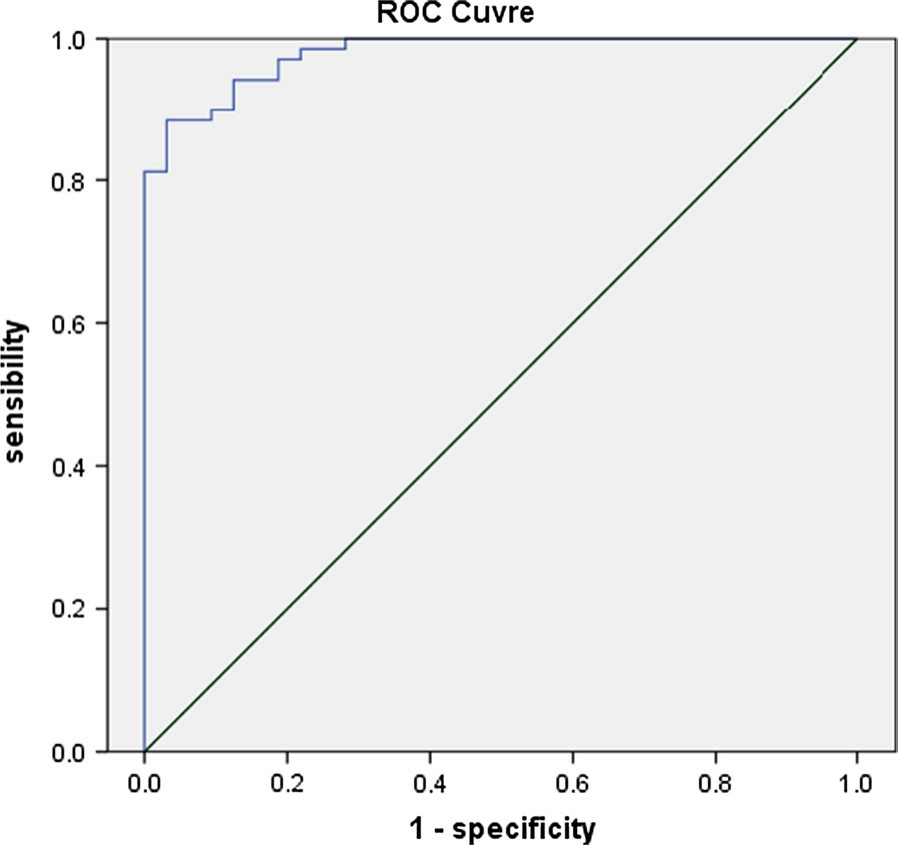
Receiver operating characteristic curves for DTF + DE + LUS + RSBI. *DE* diaphragmatic excursion, *DTF* diaphragm thickening fraction, *RSBI* rapid shallow breathing index, *LUS* lung ultrasound score

The ROC curves for RSBI, LUS, DE and DTF are shown in Figs. 1 and 2.

Weaning outcome was not accurately predicted by the RSBI area under the curve [AUC] = 0.680, 95% confidence interval (CI 0.581–0.767), *p* < 0.9). The AUCs of LUS 0.764 (CI 0.671–0.842) and DE 0.831 (CI 0.690–0.856) were higher than that of RSBI, but neither independently predicted outcome (both *p* < 0.9). The DTF AUC was 0.881 (CI 0.790–956), which was significantly greater than those of the other measures.

The best cut-off values for predicting weaning success were DTF ≥ 30%, DE ≥ 1.3 cm, LUS ≤ 11, and RSBI ≤ 102. It is worth noting that the specificity of DTF in predicting weaning outcome was higher (at 84%) than that of RBSI (53%), that of LUS (55%), and that of DE (62%), while the sensitivity at 94% was higher than that of RBSI (85%), that of LUS (71%), and that of DE (65%).

By logistic regression, we examined the potential worth of combining these variables in predicting weaning outcome. The combination of DTF ≥ 30%, DE ≥ 1.3 cm, LUS ≤ 11, and RSBI ≤ 102 showed the highest AUC (0.919), with a sensitivity of 96.0% and a specificity of 89% (Table 4, Fig. 2).

**Table 4:** Predictive accuracy of potential variables for weaning outcome

Variables	AUC	95% CI	Cut-off point	Sensitivity (%)	Specificity (%)
RSBI	0.680	0.581–0.767	102	65	53
LUS	0.764	0.671–0.842	11	71	55
DE	0.831	0.690–0.856	1.4	85	62
DTF	0.881	0.790–0.956	30	94	84
Combined model	0.919	0.850–0.963	0.23	96	89

Combined model: RSBI + LUS + DE + DTF; *AUC* area under curve, *DE* diaphragmatic excursion, *DTF* diaphragm thickening fraction, *RSBI* rapid shallow breathing index, *LUS* lung ultrasound score

## Discussion

Weaning is an important part of mechanical ventilation. There was no significant difference in the baseline data between the two groups. The mechanical ventilation time and ICU stay time of the successful weaning group were significantly shorter than those of the failed weaning group. Patients with failed weaning need to be intubated again, which increases the probability of repeated treatment and prolongs the time of mechanical ventilation and treatment. The greatest difficulty in the weaning process is the lack of one or more indicators to accurately predict whether mechanical ventilation can be successfully weaned. Ultrasound evaluation of diaphragm function has received increasing attention, including the observation of diaphragm thickening fraction (DTF), diaphragm displacement (DE) and lung ultrasound score (LUS). These indicators have certain predictive value for the weaning results of patients on mechanical ventilation. The function of diaphragmatic and respiratory muscles in elderly patients has changed due to age-related pathophysiological changes.

For the weaning of elderly patients off of mechanical ventilation, there is a lack of relevant research on the ultrasound indicators that can predict respiratory muscle function and the strength of their predictive value Previous studies have mostly focused on the evaluation of predictive indicators in patients over 18 years old, for example, Wafaa et al. [20] investigated diaphragmatic thickness as a predictor index for weaning from mechanical ventilation. While this study focused on elderly patients over 65 years old, which further clarifies the predictive value of the various indicators.

### Predictive value of the rapid shallow breathing index

One commonly used weaning indicator is RSBI, which is simple, noninvasive and repeatable. The results of this study showed that the RSBI of the successful weaning group was better than that of the failure group. ROC curve analysis showed that the failure rate of weaning was high when RBSI was greater than 102 times/(min * l). RBSI is a parameter reflecting the change in lung volume, which can reflect the contractile force of all respiratory muscle groups, and it is easy to detect clinically, so it can be used as an important reference index to evaluate the results of weaning.

### Role of lung ultrasound in predicting weaning outcomes

During the weaning process, pulmonary inflammation and cardiac insufficiency may cause loss of ventilatory volume and even lead to weaning failure. Lung ultrasound can noninvasively assess the location and extent of aeration loss, thereby assisting in the diagnosis of respiratory disorders [21]. In this study, we used LUS to quantify the degree of aeration loss before extubation. LUS was shown to be a reliable quantitative index of respiratory function and an independent predictor of weaning failure. Patients with high LUS were not suitable for weaning from mechanical ventilation. Summer et al. [14] pointed out that monitoring changes in LUS after SBT could be used to adjust treatment and to decide on the timing of extubation.

Elderly patients more often have COPD, in which case the evaluation of diaphragm function is more effective and meaningful. The reasons are as follows: it can be seen that pulmonary ultrasound has high value in the evaluation of pulmonary consolidation, but it has low value in the evaluation of chronic obstructive pulmonary disease (COPD) and other pulmonary hyperinflation disorders, and the acute exacerbation of chronic obstructive pulmonary disease is often the common cause of respiratory failure in elderly patients, so other indicators need to be added to improve the accuracy. Alvisi et al. [22] discussed the predictors of weaning outcome in chronic obstructive, pulmonary disease patients, and has shown how the failure of weaning in elderly patients with COPD is related to the decline in their lung function. Lung function is closely related to diaphragm function, so ultrasound to evaluate the diaphragm can be worthwhile.

### Role of diaphragm ultrasound in predicting weaning outcomes

The age of elderly patients is an independent factor of extubation failure. In-depth analysis revealed a decline in pulmonary function, and the decline in pulmonary function was closely related to respiratory-driven diaphragm function. Therefore, it is important to evaluate the function of the diaphragm in elderly patients. Abbas et al. [23] analyzed the role of diaphragmatic rapid shallow breathing index in predicting weaning outcome in patients with acute exacerbation of COPD, and age is an independent influencing factor of extubation failure. With increasing age, the lung organ reserve function of patients gradually decreases, and organ dysfunction is more likely to occur, leading to difficulty in extubation. At the same time, the respiratory drive reserve of the elderly is lower than that of the young, suggesting that the impairment of lung function in the elderly is related to their lower respiratory drive reserve, and the main driving force of respiration comes from the diaphragm, so the diaphragm function will affect the success rate of breathing and weaning.

The evaluation of diaphragmatic function is of great help to predict the outcome of weaning in elderly patients. The diaphragm is the most important respiratory muscle, playing a very important role in the process of spontaneous breathing. There is a correlation between mechanical ventilation time and diaphragm displacement. The more the diaphragm is displaced, the stronger the contractile function of the diaphragm, the stronger the respiratory muscle group, and the higher the weaning power are. In addition, long-term mechanical ventilation will also affect diaphragm function, resulting in diaphragm atrophy and dysfunction of diaphragm contraction, leading to weaning failure. Therefore, bedside ultrasound monitoring of diaphragm function can assess the risk of adverse events in elderly ICU patients on mechanical ventilation and predict the success rate of weaning. One traditional method for detecting diaphragm activity is transdiaphragmatic pressure measurement, but its operation is more complicated and tedious, and its clinical application is limited. Bedside ultrasound evaluation of diaphragm activity is noninvasive, safe, simple, convenient and fast, and it can also monitor the motion amplitude, thickness and contraction speed of the diaphragm in real time during breathing, making it more easily accepted by clinical practice.

The measures of diaphragmatic function include DE and DTF, which are used as ultrasound parameters to predict the success of extubation. Our study showed that sonographic indices were useful in evaluating diaphragm function to predict extubation success, supporting previous results. Diaphragm dysfunction or atrophy can be observed after mechanical ventilation for as little as 24 h [24]. Diaphragm ultrasound can be used to determine diaphragm movement to evaluate diaphragm function. Boussuges et al. [19] proposed a cut-off value for diaphragm movement of 1 cm for healthy men and 0.9 cm for healthy women. In our study, diaphragm movement < 1.3 cm was defined as hemidiaphragm dysfunction. Comparing diaphragm movement in the two groups, hemidiaphragm dysfunction was shown to be significantly associated with an increased risk of weaning failure. Therefore, US evaluation of diaphragm function may be useful to predict weaning or extubation success. Monitoring diaphragm function by US can help to detect diaphragm dysfunction earlier and aid in the decision whether to extubate.

DTF was previously explored to predict weaning from mechanical ventilation, DTF, also known as the “ejection fraction” of the diaphragm, refects active diaphragm contraction and inspiratory effort. It is associated with ICU length-of-stay, the duration of mechanical ventilation, and mortality, Ultrasonography is a widely available non-invasive tool that provides both structural and functional information about the muscle [25]. DiNino et al. [3] investigated the associations between DTF at end-expiration and end-inspiration and extubation success during either SB or PS weaning trials. They concluded that DTF measured in this way may be useful to predict extubation success or failure. Minas et al. reported a positive correlation between DTF and respiratory muscle pressure, though statistical significance was not reached.

Diaphragm indicators were better than RSBI according to the AUC and specific sensitivity. In our study, DTF was better than RSBI, LUS and DE with regard to sensitivity and specificity. The best cut-off values for predicting weaning success were DTF ≥ 30%, DE ≥ 1.3 cm, LUS ≤ 11, and RSBI ≤ 102. The specificity of DTF in predicting weaning outcome at 84% was higher than those of RBSI (53%), LUS (55%), and DE (62%), while the sensitivity at 94% was higher than those of RBSI (85%), LUS (71%), and DE (65%). The area under the DTF curve at 0.881) was significantly higher than that under the other curves. Therefore, a DTF of greater than or equal to 30% had the most accuracy for predicting weaning success, which is consistent with Ferrari et al., who reported that a cut-off value of 36% was associated with successful weaning. Samanta et al. reported that a DTF cut-off greater than or equal to 25.5% gave an AUC of 0.91 and sensitivity and specificity of 97% and 81%, respectively [26].

Despite the different cut-off values in these studies, most authors agree that DTF is a better weaning predictor than RSBI. A systematic review and meta-analysis performed by Llamas-Álvarez et al. [27] concluded that DTF is a modest predictor of weaning outcome in the general population of critically ill patients.

### Combined model for predicting weaning outcomes

As discussed, none of the individual indices measured, RSBI, LUS DE or DTF (all AUCs < 0.9), accurately predicted weaning outcome independently. However, by using logistic regression to combine them, a significantly increased AUC of 0.919, with a sensitivity and specificity of 96% and 94%, respectively, was achieved. It takes 10 min to half an hour to combine the four indicators, but it is of great value. The combined indicator can better predict extubation and is of great help to clinical practice. Diaphragm. Ultrasonography (DUS) allows serial radiation-free bedside evaluations of diaphragmatic function in critically ill patients. A combined approach consisting of a theoretical module followed by a practical training is more effective in managing acoustic windows and performing accurate measurements [28].

The present study has several limitation. Most of the studies including this study on the use of diaphragm activity and the diaphragm thickness change rate to make weaning decisions are observational studies, So, the observational design of the study has a limitation to describe the advantage of using chest ultrasound in elderly patients as compared to young patients with a considerable clinical advantage, and there is a lack of randomized controlled trials to evaluate whether weaning timing can reduce the rate of extubation failure. This study was conducted in a single centre, but multi-centre studies are needed to further confirm the results. A limitation of this study is that the sample size was small. In the future, more ICUs can be combined for multi-centre and larger-sample statistical analysis. Finally, ultrasound is an operator-dependent technique, So it depends on the technical proficiency of the operator.

## Conclusions

In conclusion, in intubated critically ill elderly patients, bedside ultrasound with assessment of diaphragm function, including DTF, DE, LUS, and RSBI, may predict weaning outcome. DTF has the highest sensitivity and specificity, making it superior to other parameters. However, the combination of these lung and diaphragm indices allows the most accurate prediction of weaning outcome. This is potentially of considerable clinical importance and merits further assessment in different patients in other settings.

## Data Availability

The datasets used and/or analysed during the current study are available from the corresponding author on reasonable request.
